# Unveiling the Hidden Biotechnological Potential of the Vermetid Gastropod *Vermetus triquetrus*: Insights into an Unexplored Marine Resource

**DOI:** 10.1007/s10126-026-10632-3

**Published:** 2026-05-28

**Authors:** A. Breves, C. Cardoso, C. Afonso, J. Matos, J. Lobo-Arteaga, C. Bartilotti, S. Sales, S. Pedro, N. M. Bandarra

**Affiliations:** 1 Portuguese Institute for the Sea and Atmosphere (IPMA, IP), Avenida Alfredo Magalhães Ramalho, 6, Algés, 1495-165 Portugal; 2 Interdisciplinary Centre of Marine and Environmental Research, CIIMAR, University of Porto, Rua dos Bragas 289, Porto, 4050-123 Portugal; 3 MARE, Marine and Environmental Sciences Centre, ARNET - Aquatic Research Network Associate Laboratory, NOVA School of Science and Technology, NOVA University Lisbon, Caparica, 2829-516 Portugal

**Keywords:** Vermetidae, Tissues, Fatty acid profile, Bioactive content, Antioxidant, Anti-inflammatory

## Abstract

The vermetid *Vermetus triquetrus*, commonly found on intertidal hard substrates in the Mediterranean Sea and the Portuguese coast, remains an underexplored marine resource. This study aimed to determine the total polyphenol content, fatty acid (FA) composition, and biological activity (antioxidant and anti-inflammatory properties) in the visceral mass and the head-foot part of this sessile gastropod from a population collected in Mira Estuary, mainland Portugal. The FA profile of the visceral mass albeit rich in saturated FA had significant levels of long-chain polyunsaturated FA, arachidonic acid (20:4 ω6), 6.3%, and eicosapentaenoic acid (20:5 ω3, EPA), 4.2% of total FA, and a non-negligible content of docosapentaenoic (22:5 ω3), 2.1%. Moreover, though the FA profile of the head-foot part was not attained, regarding other parameters, there were differences between the visceral mass and head-foot part of V. triquetrus. In fact, concerning polyphenol content, whereas the head-foot contained a total of 142 ± 14 mg GAE/100 g dw, the visceral mass reached a high level of 314 ± 90 mg GAE/100 g dw. The antioxidant activity as measured by FRAP showed a similar pattern of higher values in the visceral mass than in the head-foot, 55.6 ± 18.3 µmol Fe2+/g dw vs. 9.8 ± 0.8 µmol Fe2+/g dw. Finally, the ABTS methodology largely agreed with polyphenol levels and FRAP, being the head-foot with 1189 ± 223 µmol Trolox Eq/100 g dw less antioxidant than the visceral mass, 5511 ± 2304 µmol Trolox Eq/100 g dw. Given the presence of anti-inflammatory compounds, such as polyphenols (more abundant in the visceral mass), anti-inflammatory activity, as measured by COX-2 inhibition %, was detected, reaching more than 30% in the visceral mass. Therefore, the gastropod *V*. *triquetrus* can be a source of polyphenolic compounds, being specially its visceral mass, not only a rich source of polyphenols, but also a starting material for highly antioxidant extracts. Given the known biological activity of polyphenols and the convergence of results with the FRAP and ABTS antioxidant activity methods, further study on the specific phenolic composition and extract fractionation and refining is warranted.

## Highlights

- FA profile of the visceral mass had significant levels of LC PUFA, >10 % of total FA
- Polyphenol content was higher in the visceral mass with 314 ± 90 mg GAE/100 g dw
- FRAP antioxidant activity was higher in the visceral mass, 55.6±18.3 μmol Fe^2+^/g dw
- Anti-inflammatory activity exceeded 30% COX-2 inhibition in the visceral mass

**Graphical Abstract:**

## Graphical abstract

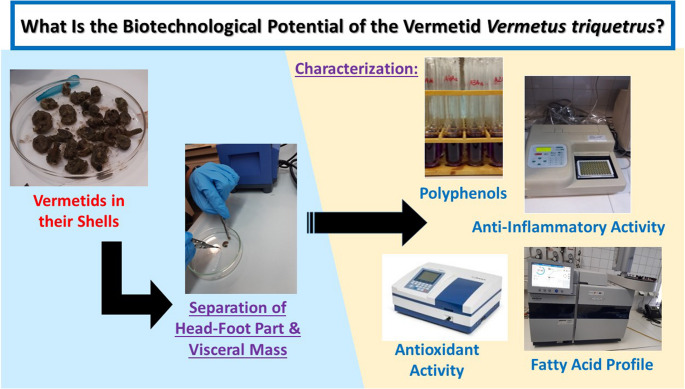

## Introduction

Vermetids are sessile and commonly gregarious gastropods characterized by irregularly uncoiled calcareous shell, cemented to or embedded in hard substrates. They usually inhabit intertidal and subtidal zones of coral reefs and rocky shores, reaching high densities in several tropical and temperate regions worldwide (Hadfield et al. 1972; Breves et al. 2017; Mercurio et al. 2025).

The wormsnails are active and passive suspension feeders and they excrete a stycky mucus net for trapping particles, which is suited to capture near-bed small planktonic organisms for food (Kappner et al. 2000; Klöppel et al. 2013). Vermetid mucus net can impact other organisms and cause strong deleterious effects on neighbouring sessile species (Brown and Osenberg 2018; Klöppel et al. 2013). The vermetid species *Ceraesignum maximum* (G. B. Sowerby I, 1825) has been documented as having negative interactions with corals, contributing to the structural homogenization of reef habitats, limiting coral growth, and increasing coral mortality (O’Malley 2017). However, little or none is known about the metabolism, biochemical composition, and biological activity of vermetids in general.

Their biological uniqueness and physiological idiosyncrasy may be coupled to hitherto unknown biochemical features, compounds, and activities. Precisely, the scarce number of studies on vermetids do not even allow to assess the biotechnological potential of this marine resource. In a unique case of a published study on the vermetid gastropod *C. maximum*, Klöppel et al. (2013) detected biologically active compounds in the mucus nets secreted by these organisms. These authors found at least two biologically active compounds exclusively accumulated by *C. maximum* in what was the first record of such bioactive properties in the Vermetidae family. More generally, gastropods and their mucus have received some scientific attention, being applications in wound healing, antimicrobials, biomaterials, and cosmetics under investigation (Fadhilah et al. 2024; Liegertová and Malý 2023).

The vermetid *Vermetus triquetrus* Bivona e Bernardi, 1832, is a common and well known species in the Mediterranean Sea (Bieler et al. 2023; Keen 1961; Scuderi et al. 2017), normally composing biogenic reefs in association with other vermetid species and crustose coralline algae (Badreddine et al. 2019; Safriel 1975). This species is also known to occur either in solitary forms or in cluster of a few individuals on the Portuguese coast in the Eastern Atlantic (Macedo et al. 1999). Its biology and detailed description as well as those of other species of the same genus are the object of rare studies (Scuderi et al. 2017), being a lineage of vermetids with a small button-like operculum (Bieler et al. 2023). Moreover, to date, there are no published studies reporting the presence of biologically active compounds in tissues or mucus net of *V. triquetrus*. Hence, its biotechnological potential is also underexplored. This has led to its inclusion in a research program within the scope of the project GENEMARE_PORTUGAL that aims at the establishment of a repository of marine biodiversity in Portuguese waters and valorization of underexplored resources. *Vermetus triquetrus* is paradigmatic of other poorly studied marine organisms. Indeed, recently, our research group found substantial anti-inflammatory activity in ethanolic extracts attained from the sea cucumbers *Holothuria (Holothuria) mammata* Grube, 1840, and *Holothuria (Roweothuria) arguinensis* Koehler & Vaney, 1906, corresponding to approximately 40% inhibition of the cyclooxygenase-2 (Carletti et al. 2022), thus highlighting the importance of targeting less studied organisms in biotechnological research.

Considering all previously mentioned aspects, an experimental design combining the extraction of components and analysis of the head-foot and visceral mass of *V. triquetrus* was set up, being determined the fatty acid composition, total phenolic content, antioxidant properties, as measured by different techniques, and anti-inflammatory activity of ethanol extracts.

## Methods

### Sample Source, Collection, and Preparation

Fresh samples of the vermetid *Vermetus triquetrus* (*n* = 10) were collected using a putty knife on intertidal rocks in Mira Estuary (37º43'39.8”N, 8º46'13.3”W) in the southern Portuguese coast in March 2025. After being collected, specimens were maintained alive with local water in individual bags and immediately brought to IPMA’s laboratory at Lisbon in order to avoid any spoilage of their tissues. In the laboratory, the specimens were washed using distilled water and carefully dissected to separate the visceral mass from the head-foot part of the animal, since these are distinct parts of the animal’s body. 

The head-foot is the region of the body with sensory and feeding functions and is generally more exposed to its surrounding environment in comparison to the visceral mass, which is normally protected by the shell. Hence, this visceral mass had remaining material from the shells, which was separated by centrifugation (5,000 × *g* at 4 °C during 15 min) in Eppendorf tubes. All samples of cleaned visceral mass and head-foot (*n* = 8 × 2) were then freeze-dried and kept frozen at -80 °C until analysis. Ethanol extracts were prepared from the freeze-dried parts and used for analysis.

### Species Identification through Integrative Taxonomy

The specimens designated as *Vermetus triquetrus* were identified using an integrative approach, combining morphological and molecular data. The morphological identification was based on soft tissue anatomy, operculum features, overall shell structure and morphology, and protoconch characteristics (Bieler 1995; Bivona-Bernardi 1832; Schiaparelli 1996; Scuderi et al. 2017), being carried out under a stereomicroscope Leica MZ12.

The molecular analysis was performed using a small fragment of tissue excised from the head-foot region of each of the specimens selected for DNA extraction (*n* = 5). The fragment of the mitochondrial cytochrome *c* oxidase subunit I gene (COI-5P) was amplified by Polymerase Chain Reaction (PCR) using puRe Taq Ready-To-Go PCR beads (Amersham Biosciences, Little Chalfont, UK) and the primer pair LoboF1 (5′-KBTCHACAAAYCAYAARGAYATHGG-3′) and LoboR1 (5′-TAAACYTCWGGRTGWCCRAARAAYCA-3′) (Lobo et al. 2013). Amplifications were carried out in a Bio-Rad thermal cycler under the following profile: initial denaturation at 94 °C for 5 min; five cycles of 30 s at 94 °C, 90 s at 45 °C and 60 s at 72 °C; followed by 45 cycles of 30 s at 94 °C, 90 s at 54 °C and 60 s at 72 °C; with a final extension of 5 min at 72 °C. Each reaction involved 1.5 µL (10 μm) of each primer and 4 µL of DNA template and was completed with sterile milli-Q^®^ water to a total volume of 25 µL. Amplification success was assessed by agarose gel electrophoresis (1.5%) in TBE buffer after staining with GreenSafe (NZYTech, Lisbon, Portugal). PCR products were purified using the quick DNA clean-up NZYGelpure kit (NZYTech, Lisbon, Portugal) and sequenced in both directions by STABVIDA (Portugal) using the BigDye Terminator v3.1 Cycle Sequencing Kit on an ABI 3730XL automated DNA sequencer (Applied Biosystems, Foster City, CA, USA).

Sequences were cleaned and edited using MEGAX to generate a consensus sequence for each specimen. The final sequences were blasted on the BOLD Identification Engine (Barcode of Life Data Systems) to get the best matches for an accurate identification.

Specimen metadata, including voucher information, images, taxonomic identification, collection details, and sequence data, were assembled in a dataset within the Barcode of Life Data Systems (BOLD; dataset code DS-VERMPT). 

### Fatty Acid Profile

The fatty acid methyl esters (FAME) were analysed by acid catalyzed transesterification as described by Bandarra et al. (1997). This method involved weighing either 10 mg of the visceral mass or 20 mg of the head-foot of *V. triquetrus* and adding 5 mL of a 5% acetyl chloride-methanolic solution (freshly prepared before use). Tubes were left to react for 1 h in a water bath adjusted to 80 °C. After cooling of the extracts, 1 mL of Milli-Q water and 2 mL n-heptane were added. Tube content was subjected to agitation and centrifuged for 3 min at 3000 × g. The organic phase was collected and filtered through anhydrous sodium sulfate. The final extract was analysed by gas chromatography in a Scion 456-GC gas chromatograph (West Lothian, UK), equipped with a capillary column DB-WAX (Agilent Technologies, Santa Clara, CA, USA), whose film thickness was 0.25 μm, length 30 m, and internal diameter 0.25 mm. The separation of the FAME was performed using helium as the carrier gas. The temperature program for the column comprised an initial phase at 180 °C, a ramp to 200 °C with a 4 °C/min gradient, a holding phase lasting 10 min at 200 °C, a second ramp to 210 °C with the same thermal gradient, and a final phase at 210 °C for 14.5 min. The identification of the FAME was achieved by the retention time with a standard mix (PUFA-3, Menhaden oil, Sigma-Aldrich) as reference. Results were expressed as percentage of the total FAME.

### Total Polyphenol Content

Phenolic compounds were extracted by 96%, v/v, ethanol, since an alcoholic extract may facilitate the upgrading of specific tissue fractions for food, cosmetic or pharmaceutical purposes. Accordingly, in order to prepare the extracts, approximately 10 mg of freeze-dried visceral mass or 40 mg of freeze-dried head-foot part was weighed, homogenized with 200 and 800 µL of ethanol 96%,v/v, respectively, using a model Polytron PT 6100 homogenizer (Kinematica, Luzern, Switzerland) at a velocity of 30,000 rpm during 1 min, and agitated for 18 h on an orbital shaker. After centrifugation (5,000 × *g* at room temperature during 20 min), the supernatant was collected through a filter to a final volume of either 200 or 800 µL depending on the initial starting weight.

The total polyphenol content was determined by the Singleton and Rossi method using the Folin-Ciocalteu reagent (Singleton and Rossi 1965). Gallic acid (GA) was used as standard and phenolic content was expressed as gallic acid equivalents (mg GAE/100 g dw) through the calibration curve of gallic acid (Sigma, Steinheim, Germany).

### Antioxidant Activity as Measured by the FRAP Method

The Ferric Ion Reducing Antioxidant Power (FRAP) method was a modified technique based on Benzie and Strain (1996) and it was applied to the same extracts used in the previous section. Results were expressed in µmol Fe^2+^ equivalent per g dw and compared with an ascorbic acid control.

### Antioxidant Activity as Measured by the ABTS Method

The ABTS (2,2’-azino-bis(3-ethylbenzothiazoline-6-sulphonic acid)) radical scavenging activity was determined using the method described by Re et al. (1999). For this, 20 µL of extract was used in triplicate. The ABTS radical scavenging activity of the samples was expressed as a percentage of inhibition as follows:

$$\%\;\mathrm{Inhibition}=(\mathrm A0-\mathrm{Asample})/\mathrm A0\times100$$

Where,

A0: Absorbance of the blank;
Asample: Absorbance of the sample.

On the basis of a trolox calibration curve, results were then expressed in µmol of trolox equivalents (Trolox Eq.) per 100 g dw.

### Anti-inflammatory Activity

The anti-inflammatory activity of the head-foot and visceral mass of *V. triquetrus* was determined in alcoholic (96%, v/v, ethanol) extracts attained from approximately 10 mg of freeze-dried visceral mass or 20 mg of freeze-dried head-foot homogenized with 100 µL and 200 µL of ethanol, respectively, using a model Polytron PT6100 homogenizer (Kinematica, Luzern, Switzerland) at a velocity of 30,000 rpm during 1 min. The extracts were subjected to heat treatment (80 °C during 1 h) and then centrifuged (3,000 × *g* at 4 °C during 10 min). The supernatant was collected and the solvent was evaporated using a vacuum rotary evaporator with the water bath temperature at 65 °C. The residue was directly dissolved in 100% dimethyl sulfoxide (DMSO) to prepare a stock preparation with a concentration of 10 mg/mL. The extract was tested at 1 mg/mL using a commercial cyclooxygenase (COX) inhibitory screening assay kit, Cayman test kit-560 131 (Cayman Chemical Company, Ann Arbor, MI, USA). A volume of 10 µL each of test extract or DMSO (blank) was used. Results were expressed as a percentage of inhibition of COX-2.

### Statistical Analysis

Normality and homogeneity of variance were tested using the Kolmogorov-Smirnov’s and Levene’s F-tests, respectively. The tissue dichotomy between visceral mass and head-foot part was the studied factor through a simple ANOVA treatment. The parametric test, Tukey Honestly Significant Difference, HSD (equal), was applied. All statistical treatment was done with STATISTICA 6, 2003 version (StatSoft, Inc., Tulsa, OK, USA), considering a significance level of (α) 0.05.

## Results

### Species Identification

The identity of the sampled specimens as belonging to the species *Vermetus triquetrus* was confirmed. Moreover, the COI-5P sequences generated from specimens collected in this study in the Mira Estuary showed low intraspecific divergence.

### Fatty Acid Profile

The fatty acid profile of the visceral mass of *Vermetus triquetrus* is shown in Table 1.

**Table 1:** Fatty acid profile (in % of total fatty acids) of the visceral mass of *Vermetus triquetrus*

Fatty Acid	(%)
14:0	1.7
16:0	22.5
18:0	14.7
Σ SFA	42.6
16:1 ω7	1.2
18:1 ω9	8.5
20:1 ω9	3.1
Σ MUFA	18.8
18:2 ω6	2.7
20:4 ω6	6.3
22:4 ω6	1.6
Σ ω6 PUFA	10.5
18:3 ω3	1.0
18:4 ω3	0.4
20:5 ω3	4.2
22:5 ω3	2.1
22:6 ω3	0.5
Σ ω3 PUFA	9.8
Σ PUFA	21.5
Σ ω3/Σ ω6	0.93

*SFA *saturated fatty acids; *MUFA *monounsaturated fatty acids; *PUFA *polyunsaturated fatty acids

The profile is characterized by a strong presence of saturated fatty acids (SFA), representing 42.6% of the total FA, followed by polyunsaturated FA (PUFA) with 21.5%, and monounsaturated FA (MUFA), 18.8%. Besides palmitic (16:0), stearic (18:0), and oleic acid (18:1 ω9), corresponding to 22.5%, 14.7%, and 8.5%, respectively, there are also relevant long-chain PUFA (LCPUFA). These are the major ω6 LCPUFA arachidonic acid (20:4 ω6, AA) with a share of 6.3% and the ω3 LCPUFA eicosapentaenoic acid (20:5 ω3, EPA) with a level of 4.2%. It is also worth mentioning a non-negligible content of 2.1% ascribed to docosapentaenoic acid (22:5 ω3, DPA). The calculated ω3/ω6 ratio did not surpass 1.0.

### Polyphenols and Antioxidant Activity

**Fig. 1:**
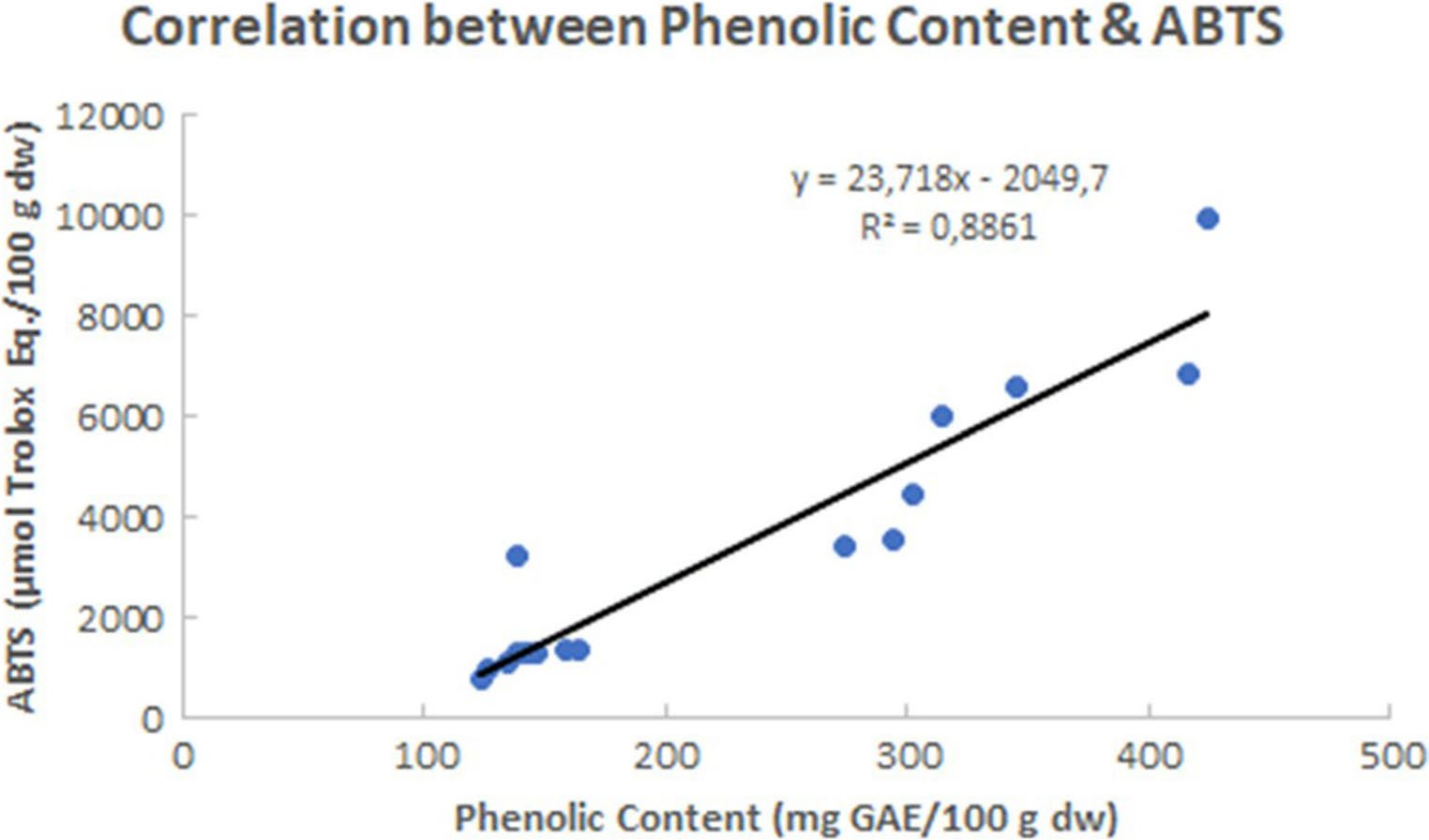
Correlation between the antioxidant results derived from the application of the ABTS method and the total polyphenol content considering all analysed specimens of *Vermetus triquetrus*

**Fig. 2:**
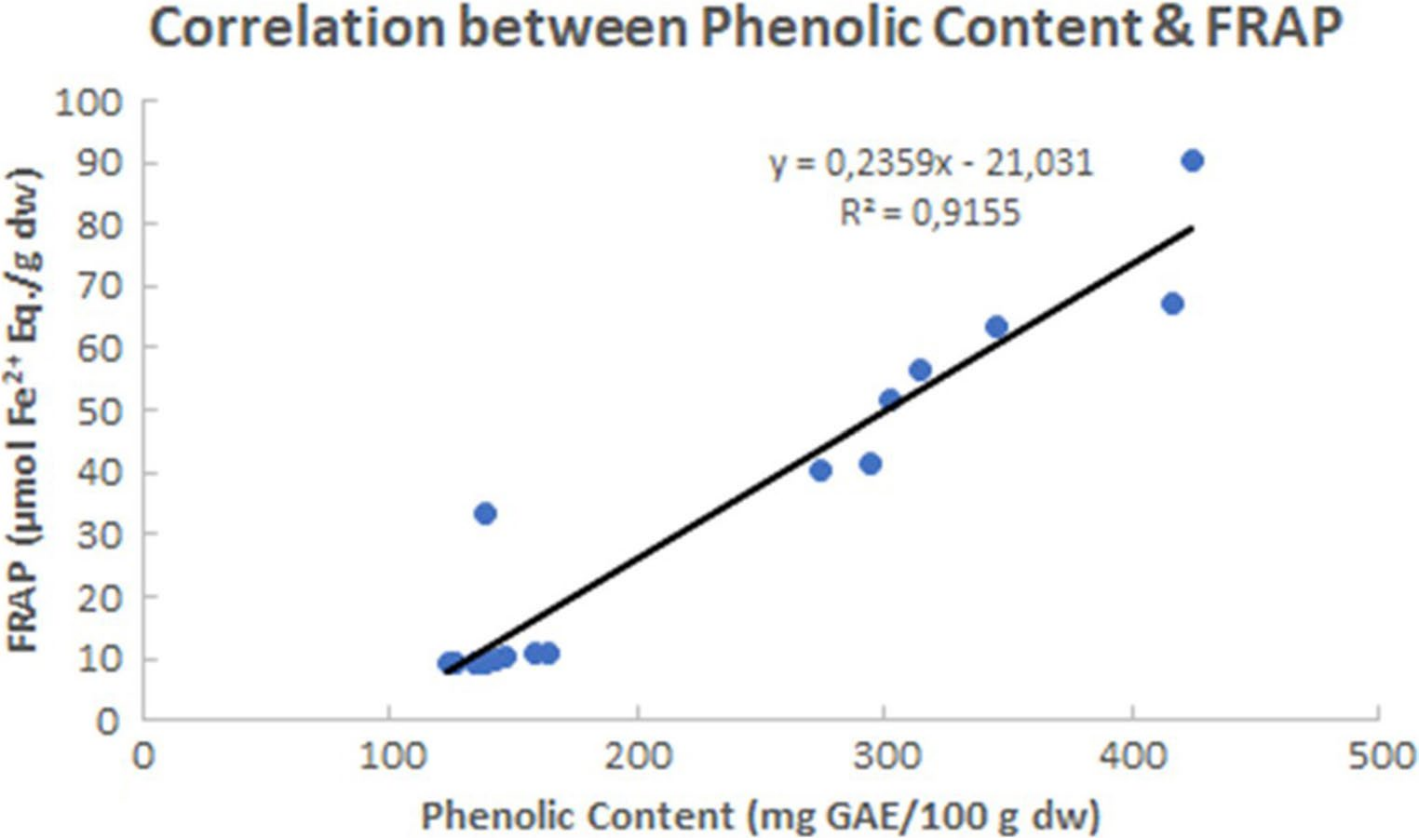
Correlation between the antioxidant results derived from the application of the FRAP method and the total polyphenol content considering all analysed specimens of *Vermetus triquetrus*

The total polyphenol content and the antioxidant activity (as measured by the ABTS and FRAP methods) in ethanol extracts of *V. triquetrus* are shown in Table 2. Relevant correlations between polyphenol content and ABTS and FRAP results are shown in Figs. 1 and 2.

**Table 2:** Total polyphenol content (in mg GAE/100 g dry weight) and antioxidant activity, as measured by ABTS (in µmol Trolox Eq./100 g dw) the FRAP (in µmol Fe^2+^/g dw), in ethanol extracts of the visceral mass and head-foot part of *Vermetus triquetrus*

Tissue	Total polyphenol (mg GAE/100 g dw)	ABTS (µmol Trolox Eq./100 g dw)	FRAP (µmol Fe^2+^/g dw)
Visceral mass	314 ± 90^**a**^	5511 ± 2304^**a**^	55.6 ± 18.3^**a**^
Head-foot	142 ± 14^**b**^	1189 ± 223^**b**^	9.8 ± 0.8^**b**^

Values are presented as average±standard deviation. Different lowercase letters within a column correspond to statistical differences (*p* < 0.05) between anatomical parts

Firstly, regarding the phenolic content, there were differences between the visceral mass and head-foot part of *V. triquetrus*. In fact, whereas the head-foot contained a total of 142 ± 14 mg GAE/100 g dw, the visceral mass reached a high level of 314 ± 90 mg GAE/100 g dw. The high standard deviation resulted from a substantial variability between individuals, which may also be affected by digestive contents at the moment of capture. On the other hand, the antioxidant activity as measured by FRAP showed a similar pattern of higher values in the visceral mass than in the head-foot, 55.6 ± 18.3 µmol Fe^2+^/g dw vs. 9.8 ± 0.8 µmol Fe^2+^/g dw. Finally, the ABTS methodology largely agreed with polyphenol levels and FRAP, being the head-foot part less antioxidant than the visceral mass.

### Anti-inflammatory Activity

The anti-inflammatory activity (expressed as percentage of inhibition of COX-2) in the ethanol extracts of the vermetid gastropod species *V. triquetrus* is displayed in Table 3.

**Table 3:** Anti-inflammatory activity (% inhibition of COX-2) in ethanol extracts of the visceral mass and head-foot part of *Vermetus triquetrus*

Tissue	Anti-inflammatory activity (% COX-2 inhibition)
Visceral mass	34.6 ± 22.0^**a**^
Head-foot	13.2 ± 0.4^**a**^

Values are presented as average±standard deviation. Different lowercase letters within a column correspond to statistical differences (*p* < 0.05) between anatomical parts

In accordance to the presence of anti-inflammatory compounds, such as polyphenols (more abundant in the visceral mass), anti-inflammatory activity, as measured by COX-2 inhibition, was detected. It was higher in the visceral mass, thereby surpassing 30% of COX-2 inhibition, a significant value.

## Discussion

### Species Identification

Regarding the low intraspecific divergence in the COI-5P sequences, a comparable pattern was observed when these sequences were aligned with the only publicly available COI-5P record of *Vermetus triquetrus* (Lobo et al. 2013), obtained from a nearby locality in 2009 (BOLD sample ID GTALE008-09; BIN BOLD: ABV4357; GenBank accession number KF369193), with a sequence similarity of 99.8%.

A detailed morphological redescription of the vermetid *V. triquetrus* is necessary and is currently under preparation by our research group.

### Fatty Acid Profile

With respect to the fatty acid profile, this was a preliminary study that was hampered by the exiguous amount of sample, which, in the case of the head-foot part, did not allow profiling as a result of being below the limit of quantification. In any case, since to the best of the authors’ knowledge, the fatty acid profile of *V. triquetrus* was never previously studied, the preliminary results of the visceral mass deserve analysis. It is possible the comparison to the FA profile of marine gastropods, such as the visceral material from *Turbo cornutus* [Lightfoot], 1786, thereby revealing some striking similarities with the visceral mass of *V. triquetrus* (Saito and Aono 2014). In fact, the EPA content in *T. cornutus* viscera triacylglycerols (TAG, reserve lipids) was identical to that of *V. triquetrus* viscera, 4.2 ± 0.3% of total FA, and the DPA content was quite similar, 3.4 ± 0.1%. The docosahexaenoic acid (22:6 ω3, DHA) in the *T. cornutus* viscera TAG was also very low, reaching 0.6 ± 0.0% (Saito and Aono 2014). On the other hand, the AA in the viscera TAG of this marine gastropod, 8.9–12.9%, is higher than in the viscera of the vermetid (Saito and Aono 2014). The overall distribution between SFA, MUFA, and PUFA in the visceral mass of *V. triquetrus* has similarities with other marine gastropod species, *Buccinanops cochlidium* (Dillwyn, 1817) and *Trophon geversianus* (Pallas, 1774), characterized by a predominance of SFA, followed by PUFA and MUFA (Bigatti et al. 2018). In addition, the fact that *V. triquetrus* was sampled from an intertidal zone with strong exposure to UV irradiation (Davis et al. 2013) may underlie the large share of SFA, given the relative chemical stability of these FA against UV-induced oxidation (Guihéneuf et al. 2010). Seasonality may also be influential. Fokina et al. (2018), while studying the blue mussel *Mytilus edulis* Linnaeus, 1758, a bivalve mollusc in an intertidal habitat, observed that the accumulation of ω3 and ω6 PUFA (in phospholipids present in the gills) was higher in November (lower temperature) and lower in May (higher temperature), explaining this by the homeoviscous adaptation to temperature and reserves depletion in Winter. Thus, *V. triquetrus* collected in March may have an FA composition that is a result of UV irradiation, temperature, reserve depletion, and also reproduction phase (Calvo and Templado 2004). Hence, this first set of FA results in a vermetid sample warrants further study.

### Polyphenols and Antioxidant Activity

Concerning all studied parameters, there was a large significant difference in the concentration of the polyphenols, as bioactive compounds, and biological activity between visceral mass and the head-foot. This may be due to a higher activity of enzymes and highly active biosynthetic routes in visceral organs as in other mollusc viscera (Rivera-Pérez et al. 2023). There was also high variability within the visceral mass results. Since the vermetid gastropods have not been comprehensively studied, comparisons can be made to other gastropod groups (Mu’minun et al. 2025; Salas and Chakraborty 2020) and different marine organisms, such as tunicates and sea cucumbers (Carletti et al. 2022).

As to total polyphenol content, in the marine gastropods *Chicoreus ramosus* (Linnaeus, 1758) and *Babylonia spirata* (Linnaeus, 1758), it reached very high levels, particularly in ethyl acetate-methanol extracts, 4651–7362 mg GAE/100 g of extract (Salas and Chakraborty 2020). Since results of *V. triquetrus* are per 100 g dw of biomass, a direct comparison is not possible. For the sea hare *Dolabella auricularia* ([Lightfoot], 1786), a total polyphenol content of approximately 280 mg GAE/100 g was displayed by the ethanol extract attained from its ink (Tayone and Del Rosario 2017). Though the value is similar to the vermetid visceral mass, it is a very specific tissue and in the fresh form (Tayone and Del Rosario 2017). Considering the black-footed abalone *Haliotis iris* Gmelin, 1791, a study on extractability and activity (Mohammadi et al. 2022) revealed a wide range of total polyphenol levels from 500 to 3000 mg GAE/100 g dw in this gastropod. These levels surpass the results obtained for the visceral mass of *V. triquetrus*, but it involved an optimized subcritical water extraction instead of a direct solid-liquid extraction with ethanol as done in the case of the vermetid. Beyond gastropods, there is a recent study (Carletti et al. 2022) that used the same methodology and encompassed three sea cucumber species, *H. mammata*, *Holothuria forskali* Delle Chiaje, 1824, and *H. arguinensis*, having determined phenolic levels in the 48–84 mg GAE/100 g dw interval in the whole biomass of these species, which is well below the values observed in the head-foot and, even more, in the visceral mass of *V. triquetrus*.

The antioxidant activity, comprising both ABTS and FRAP techniques, delivered a set of consistent results. Regarding ABTS, a comparison to other marine gastropods is possible and, for instance, the extracts of edible parts from *C. ramosus* and *B. spirata* have been shown to be antioxidant with higher ABTS antioxidant activity in ethyl acetate-methanol extracts than in chloroform extracts, thereby indicating that antioxidant compounds are more polar and less lipophilic (Salas and Chakraborty 2020). Jia et al. (2024) reported on the antioxidant activity of abalone visceral peptides and determined ABTS levels of nearly 1000 µmol Trolox Eq./100 g of dry sample, which is approximately the value measured in the head-foot part and much lower than the value for the visceral mass of *V. triquetrus*. Since Jia et al. (2024) worked with peptides with more exposed antioxidant active sites as a result of protease action, this comparison highlights the strong antioxidant potential of the visceral mass in the vermetid. Furthermore, Mu’minun et al. (2025) also studied an abalone, *Haliotis asinina* Linnaeus, 1758, and found that the visceral organ extract —using 96%, v/v, ethanol, just as in the current study— derived from this gastropod had an IC_50_ value of 22.48 µg/ml, an antioxidant power deemed substantial, comparable to that of the specific antioxidant polyphenol quercetin. In addition, recent studies have highlighted the potential of visceral peptides from gastropods as a possible source for the production of functional foods due to their strong antioxidant activity (Liu et al. 2023). In holothurians extracted with ethanol and analysed by the same ABTS technique, Carletti et al. (2022) observed low antioxidant levels, < 200 µmol Trolox Eq./100 g dw. Only in the colonial tunicates *Aplidium* sp. and *Botrylloides diegensis* Ritter & Forsyth, 1917, ABTS yielded values between 5000 and 6000 µmol Trolox Eq./100 g dw (Carletti et al. 2022), thus matching the level in the visceral mass of *V. triquetrus*.

Concerning FRAP, a comparison to literature on antioxidant activity in other marine organisms points to a high antioxidant activity in the studied tissues of *V. triquetrus*, since, for instance, Carletti et al. (2022) measured FRAP values of 6.2–8.9 µmol Fe^2+^/g dw in the aqueous extracts from sea cucumbers and even lower in ethanol extracts. These are whole biomass values that are relatively near to the low antioxidant FRAP levels in the head-foot of *V. triquetrus* (Table 2). On the other hand, the tunicate *B. diegensis* displayed high FRAP values in both aqueous and ethanol extracts that were in the vicinity of 55 µmol Fe^2+^/g dw, quite similar to the vermetid visceral mass.

The importance of studying separately the visceral mass is corroborated by studies on distinct species of gastropods. Namely, in the sea snail *T. cornutus*, the visceral mass had higher antioxidant activity than the muscle (Kang et al. 2021). Indeed, the hydrogen peroxide scavenging activity surpassed 80% in enzyme-assisted viscera extracts, but did not reach 60% activity in muscle extracts (Kang et al. 2021).

It is well known that the total polyphenol content may correlate with the antioxidant activity in the extracts, since the chemical properties of phenolic compounds enable a strong antioxidant action whenever high levels are found (Dimitrios 2006). Therefore, correlations between total polyphenol content and either ABTS or FRAP were investigated. The obtained correlations were relatively strong with an R^2^ of 0.89 for ABTS and 0.92 for FRAP (Figs. 1 and 2). Hence, there is a high level of agreement between two different techniques that measure dissimilar aspects of the antioxidant activity, both pointing to a key role of phenolic compounds in supporting a high antioxidant activity in the ethanol extracts from *V. triquetrus*. In this context, it should be noted that whereas FRAP test is related to the presence of potential electron donors, the ABTS assay is used to assess radical scavenging capacity for both hydrophilic and lipophilic compounds/samples, since the ABTS radical is soluble in aqueous and organic solvents (Csepregi et al. 2016; Magalhães et al. 2008; Re et al. 1999). In the case of *V. triquetrus*, it seems that phenolic compounds operate as electron donors and are effective radical scavengers, being possible that specific phenolic compounds have a larger contribution to the observed antioxidant properties than others. In the sea cucumber *Holothuria scabra* Jaeger, 1833, Wulandari et al. (2022) found that antioxidant activity, such as measured by FRAP, was related to the total flavonoid (a particular class of phenolic compounds) content. Therefore, a detailed profiling of the phenolic compounds in *V. triquetrus*, especially in the visceral mass, should be the subject of future studies.

An issue affecting both polyphenol content and antioxidant activity levels was the high variability in the visceral mass. A strong inter-individual variability may be more strongly expressed in the visceral mass due to the dependence of this organism part on the ingested food and digestive process. Given the fact that this organism is an active and passive suspension feeder targeting near-bed small planktonic organisms (Kappner et al. 2000; Klöppel et al. 2013), its visceral mass composition may be affected by the plankton ingested immediately before sampling. Moreover, since sampling occurred in March, there is extensive overlap with the more intense period of the *V. triquetrus*’ reproductive cycle (Calvo and Templado 2004). Indeed, according to these authors, the highest numbers of egg capsules and the period of greatest reproductive activity of females corresponds to Spring to early Summer. In males, spermatogenesis has been reported to occur more or less continuously throughout the year, being mature males always present (Calvo and Templado 2004). Therefore, inter-individual variability may result from differences between males and females as well as between females transitioning from a less active to more active reproductive stage. The formation of egg capsules represents a large investment of nutrients and energy and may affect more intensely the composition of the visceral mass. Hence, the seasonality of the composition and biological activity in *V. triquetrus* also warrants further research.

### Anti-inflammatory Activity

The ethanol extracts of the two anatomical components of *V. triquetrus* had some variability in the anti-inflammatory activity levels, a variation of approximately 20% COX-2 inhibition. This departs from values determined by Carletti et al. (2022) in aqueous extracts from holothurians *H. mammata*, *H. forskali*, and *H. arguinensis* subjected to the same in vitro technique as in the present study for evaluation of the anti-inflammatory activity. There was no anti-inflammatory activity in the aqueous extracts (Carletti et al. 2022). However, the ethanol extracts of these holothurian species had substantial COX-2 inhibition, in the 16–42% interval, thereby partially superimposing with the interval for the studied anatomical parts of the vermetid. Other teams of researchers have also examined the anti-inflammatory activity in extracts of similar understudied marine organisms, but through different methods, such as nitric oxide production inhibition (Pranweerapaiboon et al. 2020), induced inflammation in THP-1 cells (Kareh et al. 2018), and in vivo experimentation (Moradi et al. 2020).

The substantial anti-inflammatory activity in a specific anatomical part, especially the visceral mass, may be due to either specific compounds biosynthesized and accumulated in that part or to the microbiome constitution of the animal gut (Yamazaki et al. 2020) influencing local biochemistry. However, the corresponding bioactive compounds underlying the determined anti-inflammatory activity in the visceral mass of *V. triquetrus* were not found. The polyphenols previously addressed as antioxidants and viable promoters of the ABTS and FRAP antioxidant activity are also a possibility in the case of anti-inflammatory activity (Pereira and Cotas 2023). Indeed, marine polyphenolic compounds can inhibit pro-inflammatory signalling routes, such as nuclear factor-kappa B (NF-κB) and mitogen-activated protein kinases (MAPKs). These compounds also suppress the production of pro-inflammatory cytokines, such as IL-6, TNF-α, and chemokines. It should also be noted that oxidative stress is closely associated to inflammation and mitigated by polyphenols. Furthermore, polyphenols have the potential to suppress key enzymes, including inducible nitric oxide synthase (iNOS) and COX-2. The effect on these enzymes leads to a fine-tuning of the levels of inflammatory mediators, such as nitric oxide (NO) and prostaglandin E-2 (PGE-2) (Degl’Innocenti and Vasarri 2021; Nagahawatta et al. 2024). If the polyphenol anti-inflammatory action hypothesis is confirmed, it would be justified to proceed to a component fractionation guided by the anti-inflammatory activity in order to isolate bioactive phenolic molecules (Rosa et al. 2023).

Furthermore, to the best of the authors’ knowledge, no previous study on the anti-inflammatory activity of polyphenols in vermetids has been performed. The nearest instance of anti-inflammatory properties is found in marine gastropods (Shin et al. 2022). Namely, brownish abalone (*Haliotis discus hannai* Ino, 1953) viscera were extracted with acetone and fractionated by different six acetone/hexane ratios, being one of the fractions a potent inhibitor of lipopolysaccharide-induced NO production without cytotoxicity (Shin et al. 2022). Western blot analysis revealed that this fraction down-regulated the activation of NF-κB and MAPK as well as inflammatory enzymes, comprising iNOS and COX-2, suggesting that it may be an effective therapeutic and functional material for treating inflammatory disorders (Shin et al. 2022).

## Conclusions

This is a relevant study and the first report in what concerns composition and biological activity in tissues of a vermetid species and, in particular, belonging to *V. triquetrus* —as ascertained by molecular biology species identification—, including a differentiation between two main anatomical parts of the animal. The FA profile of the visceral mass albeit rich in SFA had significant levels of LCPUFA, arachidonic acid (20:4 ω6), 6.3%, and eicosapentaenoic acid (20:5 ω3, EPA), 4.2% of total FA, and a non-negligible content of docosapentaenoic (22:5 ω3), 2.1%. Concerning polyphenol content, whereas the head-foot part contained a total of 142 ± 14 mg GAE/100 g dw, the visceral mass reached a high level of 314 ± 90 mg GAE/100 g dw. The antioxidant activity as measured by FRAP showed a similar pattern of higher values in the visceral mass than in the head-foot, 55.6 ± 18.3 µmol Fe^2+^/g dw vs. 9.8 ± 0.8 µmol Fe^2+^/g dw. Finally, the ABTS methodology largely agreed with polyphenol levels and FRAP, being the head-foot with 1189 ± 223 µmol Trolox Eq/100 g dw less antioxidant than the visceral mass, 5511 ± 2304 µmol Trolox Eq/100 g dw. Given the presence of anti-inflammatory compounds, such as polyphenols (more abundant in the visceral mass), anti-inflammatory activity, as measured by COX-2 inhibition %, was detected, reaching more than 30% in the visceral mass. The observed differences between the visceral mass and the head-foot should be further investigated. *V. triquetrus* is undoubtedly a source of polyphenolic compounds, being specially its visceral mass, not only a rich source of polyphenols, but also a starting material for highly antioxidant extracts. Through bio-guided fractionation it may be possible to isolate bioactive molecules and, while contributing to a biorefinery approach, envisage high value-added applications.

## Data Availability

Research data are made available if requested.
